# Cartilage progenitor cells combined with PHBV in cartilage tissue engineering

**DOI:** 10.1186/s12967-019-1855-x

**Published:** 2019-03-29

**Authors:** Ke Xue, Xiaodie Zhang, Zixu Gao, Wanyao Xia, Lin Qi, Kai Liu

**Affiliations:** 1 Department of Plastic and Reconstructive Surgery, Shanghai 9th People’s Hospital, Shanghai Key Laboratory of Tissue Engineering, Shanghai Jiao Tong University School of Medicine, 639 Zhi Zao Ju Road, Shanghai, 200011 People’s Republic of China; 2 The Second Clinical Medical College of Nanchang University, Jiangxi Medical College, Nanchang University, No. 461, Bayi Avenue, Nanchang, 330006 China; 3 Department of Radiology, Huadong Hospital, Fudan University, 221 West Yan-an Road, Shanghai, 200040 China

**Keywords:** BMSCs, Chondrocytes, Cartilage progenitor cells, Fibronectin, Chondrogenic differentiation

## Abstract

**Background:**

Bone marrow-derived stem cells (BMSCs) and chondrocytes have been reported to present “dedifferentiation” and “phenotypic loss” during the chondrogenic differentiation process in cartilage tissue engineering, and cartilage progenitor cells (CPCs) are novel seeding cells for cartilage tissue engineering. In our previous study, cartilage progenitor cells from different subtypes of cartilage tissue were isolated and identified in vitro, but the study on in vivo chondrogenic characteristics of cartilage progenitor cells remained rarely. In the current study, we explored the feasibility of combining cartilage progenitor cells with poly(3-hydroxybutyrate-co-3-hydroxyvalerate) (PHBV) to produce tissue-engineered cartilage and compared the proliferation ability and chondrogenic characteristics of cartilage progenitor cells with those of bone marrow-derived stem cells and chondrocytes.

**Methods:**

These three cells combined with PHBV were cultured in vitro for 1 week without chondrogenic induction and then transplanted subcutaneously into nude mice for 6 weeks. The cell-PHBV constructs were evaluated by gross observation, histological staining, glycosaminoglycan content measurement, biomechanical analysis and RT-PCR.

**Results:**

The chondrocyte-PHBV constructs and CPC-PHBV constructs became an ivory-whitish cartilage-like tissue, while the BMSC-PHBV constructs became vascularized 6 weeks after the subcutaneous implantation. Histological examination showed that many typical cartilage structures were present in the chondrocyte group, some typical cartilage structures were observed in the CPC group, while no typical cartilage structures were observed in the BMSC group.

**Conclusions:**

Cartilage progenitor cells may undergo chondrogenesis without chondrogenic induction and are better at chondrogenesis than BMSCs but worse than chondrocytes in the application of cartilage tissue engineering.

## Background

The repair of cartilage defects caused by trauma, tumour and congenital factors remains a major challenge for plastic and orthopaedic surgeons because cartilage has a low spontaneous repair and regeneration capacity. Several strategies have been developed to restore and repair cartilage defects, such as microfracture, abrasion chondroplasty, cartilage scraping, and transplantation of the perichondrium, periosteum, and cartilage [1]. However, these strategies generate tissue that cannot substitute for native cartilage, and the results of currently available therapies are far from satisfactory [2].

Autologous chondrocyte implantation (ACI) presents encouraging results [3]. Kuroda et al. demonstrated that a three-dimensionally structured autologous chondrocyte implant was effective in repairing cartilage defects in a rat model of anterior cruciate ligament-induced osteoarthritis (OA) [4]. Over the past 20 years, combining biomaterial scaffolds and cell sources to induce cartilage regeneration has emerged as a promising new strategy [5]. Fulco et al. isolated chondrocytes from the nasal septum and engineered autologous nasal cartilage tissues to repair cartilage defects after skin cancer excision [6]. The cutaneous sensitivity and structural stability of the reconstructed area were clinically satisfactory, with adequate respiratory function [6]. However, cell-based therapy, including chondrocyte expansion in vitro, results in loss of the chondrocyte phenotype and ageing [7].

Owing to its multipotent differentiation potential and high proliferative potential, bone mesenchymal stem cell (BMSC)-based cartilage tissue engineering and cartilage regenerative medicine offer a promising strategy for treating cartilage defects [8]. Jia et al. demonstrated that differentiated BMSCs combined with an oriented scaffold can successfully repair full-thickness articular cartilage defects in rabbits and produce cartilage with enhanced biomechanical properties [9]. However, an increasing number of studies have demonstrated that although chondrogenically differentiated BMSCs show chondrogenic potential in advance, these cells tend to eventually vascularize or ossify, undergo terminal chondrocyte differentiation and are replaced by osseous tissue, which indicates that the chondrogenic differentiation of BMSCs represents a transient state only [10].

Recently, chondrogenic stem/progenitor stem cells derived from cartilage tissue were isolated and identified. In our previous study, we isolated cartilage stem/progenitor stem cells in a pig model by a differential adhesion assay to fibronectin, and evaluated the stemness of the cartilage stem/progenitor stem cells [11, 12]. However, there is only few research concerning the chondrogenic characteristics of cartilage progenitor cells (CPCs) in vivo [13]. Several studies have used PHB and PHBV as biomaterials for cartilage tissue engineering [14–16], and we used PHBV as a scaffold for cartilage tissue engineering and showed that PHBV scaffolds have the potential to be used as chondrocyte carriers for cartilage engineering in our previous study [17]. In the current study, we explored the feasibility of combining CPCs with poly(3-hydroxybutyrate-co-3-hydroxyvalerate) (PHBV) to produce tissue-engineered cartilage and compared the in vitro proliferation ability and in vivo chondrogenic characteristics of CPCs with those of BMSCs and chondrocytes.

## Materials and methods

All experimental protocols involving animal tissues and cells were approved by the Ethics Committee of Shanghai Jiao Tong University School of Medicine.

Chondrocytes and CPCs were harvested from swine articular cartilage tissue via differential adhesion to fibronectin in vitro, as described previously [18, 19]. The obtained articular cartilage tissues were minced into 1 mm^2^ pieces and then washed in sterile PBS and chloromycetin thrice. The cartilage tissue was digested in high-glucose DMEM containing 0.1% collagenase type 2 in a 37 °C shaking water bath for 6–8 h. Then, the suspension was filtered through a 200-μm filter to remove undigested particles, and chondrocytes at a density of 4000 cells/ml were seeded onto 10-cm plastic dishes (treated with 10 μg/ml fibronectin overnight) at 37 °C for 20 min in low-glucose DMEM. After 20 min, non-adherent cells and media were removed, and low-glucose DMEM containing 10% FBS was added to the plates. The adherent cells were cultured for 7–14 days until the cells reached 80–90% confluence. The cells were then digested with 0.25% trypsin plus 0.02% EDTA (Invitrogen) and sub-cultured into new dishes at a density of 2 × 10^4^ cells/cm^2^.

Swine bone marrow was obtained from the posterior superior iliac crest of newborn pigs as described previously [20]. Low-glucose DMEM supplemented with 10% FBS was added to the aspirate (1:1) and loaded over Percoll (Sigma, St. Louis, Mo., USA) for density gradient centrifugation. Mononucleated cells were harvested from the interface after centrifugation at 3000 rpm for 10 min and then washed twice with PBS. Cells were re-suspended in low-glucose DMEM containing 10% FBS, plated into 100-mm culture dishes at a density of 2 × 10^5^ cells/cm^2^ and incubated at 37 °C in an atmosphere of 5% CO_2_ in air. Non-adherent cells were removed by a medium change after 24 h. Adherent cells were cultured for 7–14 days until cells reached at least 80–90% confluence. The cells were then digested with 0.25% trypsin plus 0.02% EDTA and sub-cultured in a 100-mm culture dish at a density of 2.5 × 10^4^ cells/cm^2^. The medium was changed twice a week until the cells were 80–90% confluent.

### Cell proliferation in vitro

The cell proliferation rate was assessed with a cell counting kit (CCK)-based colorimetric assay (CCK-8; Dojindo China Co., Ltd.). Chondrocytes, BMSCs, and CPCs re-suspended in 100 μl of DMEM containing 10% FBS were seeded at a density of 1000 cells/well in 96-well plates and cultured for 1 day, 3 days, 5 days, and 7 days. Before every test, 10 μl of CCK-8 solution was added to each well and incubated for 4 h. Then, the absorbance of the supernatant was measured spectrophotometrically at 450 nm, and the test was performed in triplicate.

### Preparation of PHBV scaffolds and cell-scaffold constructs

PHBV scaffolds were prepared using a solvent casting-particulate leaching method as described previously [17]. Scaffolds cut into the shape of cylinder with the same size (5 mm diameter, 2 mm thick) were used in the study. The PHBV scaffolds were first evaluated by optical microscopy and then examined with a scanning electron microscope (SEM; EPMA-8705QH2, Shimadzu, Japan) after being coated with gold as described previously [17], and the SEM examination was performed at an accelerating voltage of 20 kV.

Chondrocytes, BMSCs, and CPCs at passage 2 (2.5 × 10^6^ in 40 µl) were then evenly dropped onto each scaffold. After 4–6 h of incubation to allow adequate adhesion of the cells to the scaffold, low-glucose DMEM containing 10% FBS was added to immerse the cell-scaffold construct. The constructs were then cultured in an incubator at 37 °C with 5% CO_2_ and 95% humidity. After 1 week of in vitro culture, the constructs were implanted subcutaneously into nude mice and harvested at 6 weeks post implantation.

### Wet weight and volume measurement

The weight and volume measurement of in vivo engineered tissue were measured 6 weeks after implantation. The wet weight of each specimen was measured using an electronic balance, and the diameter and thickness of each specimen were measured with vernier callipers.

### Glycosaminoglycan (GAG) and total collagen

Six weeks after implantation, the glycosaminoglycan (GAG) content of the specimens was assayed by Alcian blue colorimetric analysis as previously described [21]: the specimens were ground to obtain a protein solution. A series of reagents was added step by step to ensure specific binding of Alcian blue to polysulfated GAG molecules in cartilage. All GAGs were precipitated specifically in guanidine-HCl using a low pH in combination with detergent and a high salt concentration. The precipitate was then dissolved in a mixture of guanidine-HCl and propanol. For quantification, absorbance was recorded using a microplate reader with a 600-nm filter, and a linear standard curve between 0.5 and 20 mg was generated by adding known amounts of proteoglycans.

The total collagen content was analysed according to previously described methods [22]. Six weeks after in vivo transplantation, cell-scaffold constructs were rinsed with PBS and then lyophilized. Subsequently, the dry mass of lyophilized samples was measured and then hydrolysed in 6 N HCl, and the hydroxyproline concentration was analysed to determine collagen content.

### Histology and immunohistochemistry

The samples harvested 6 weeks after implantation were prepared for histological and immunohistochemical examination to evaluate chondrogenesis. The specimens were first fixed in buffered 10% formalin in PBS for 4–6 h, embedded in paraffin and then cut into 5-μm sections. The sections were stained with haematoxylin and eosin (HE), safranine-*O* and type II collagen (COL II) to evaluate the histological structure and cartilage matrix deposition in engineered tissue. COL II expression was detected using a mouse anti-human COL II monoclonal antibody (1: 100 in PBS; Santa Cruz, Santa Cruz, Calif., USA) and a horseradish peroxidase-conjugated anti-mouse secondary antibody (1: 200 in PBS; Santa Cruz) followed by colour development with diaminobenzidine tetrahydrochloride (Santa Cruz).

### GAG, total collagen and biomechanical analysis

A biomechanical analyser (Instron, Canton, Mass., USA) was used for biomechanical tests. As previously described [23], a constant compressive strain rate of 1 mm/min was applied until a maximal force of 100 N was achieved; thus, a force–displacement curve was obtained. The compressive modulus of the tested tissue was calculated from the force–displacement curve.

### Real-time quantitative polymerase chain reaction

The samples were harvested 6 weeks after in vivo implantation, total RNA was extracted from each specimen, and cDNA was obtained by reverse transcription (RT) according to previously described methods [24], the gene expression was evaluated by real-time quantitative PCR analysis with the brilliant SYBR green qPCR kit (Stratagene, USA). The PCR reactions were performed using a real-time PCR detection system (Bio-Rad Laboratories) and thermo cycler conditions following suggestions of the manufacturer. The relative gene expression levels were determined using the 2ΔΔCT method. Aggrecan, collagen II, and sox-9, as well as VEGF, were used to evaluate chondrogenic differentiation. The primers used in this study are shown in Table 1. The β-actin mRNA level was quantified as an internal control. The experiments were repeated at least three times.

**Table 1:** Primer sequences for PCR

Gene	Accession numbers	Primer	Product (bp)
Aggrecan	NM_001135	Sense 5′-GGGGAATCTTCTGGCATTAA-3′	381
		Antisense 5′-CGTTGGAGCCTGGGTT-3′	
SOX-9	NM_000346.4	Sense 5′-GGCTCGGACACAGAGAACAC-3′	195
		Antisense 5′-GTGCGGCTTATTCTTGCTCG-3′	
COL II a1	NM_001844.5	Sense 5′-TGCTGCTGACGCTGCTC-3′	294
		Antisense 5′-GTTCTCCTTTCCTGTCCCTTTG-3′	
VEGF	NM_001025366.2	Sense 5′-CATCTTCAAGCCGTCCTGTGT-3′	142
		Antisense 5′-TCCTATGTGCTGGCCTTGGT-3′	
β-Actin	NM_001101.5	Sense 5′-ACATCAAGGAGAAGCTCTGCTACG-3′	366
		Antisense 5′-GAGGGGCGATGATCTTGATCTTCA-3′	

### Enzyme-linked immunosorbent assay

The VEGF content in three groups 6 weeks after in vivo implantation was quantified using ELISA kits (R&D Systems) according to the manufacturer’s instructions as previously described [25], and the plates were incubated with 100 μl of VEGF standards and diluted samples. The intensities were determined at 450 nm using a microplate reader (Thermo Scientific, USA). The test was performed in triplicate.

### Statistical analysis

Statistical evaluations were performed using an ANOVA followed by post hoc analysis. A p value less than 0.05 was considered statistically significant.

## Results

### Culture of CPCs, BMSCs, and chondrocytes in vitro

**Fig. 1:**
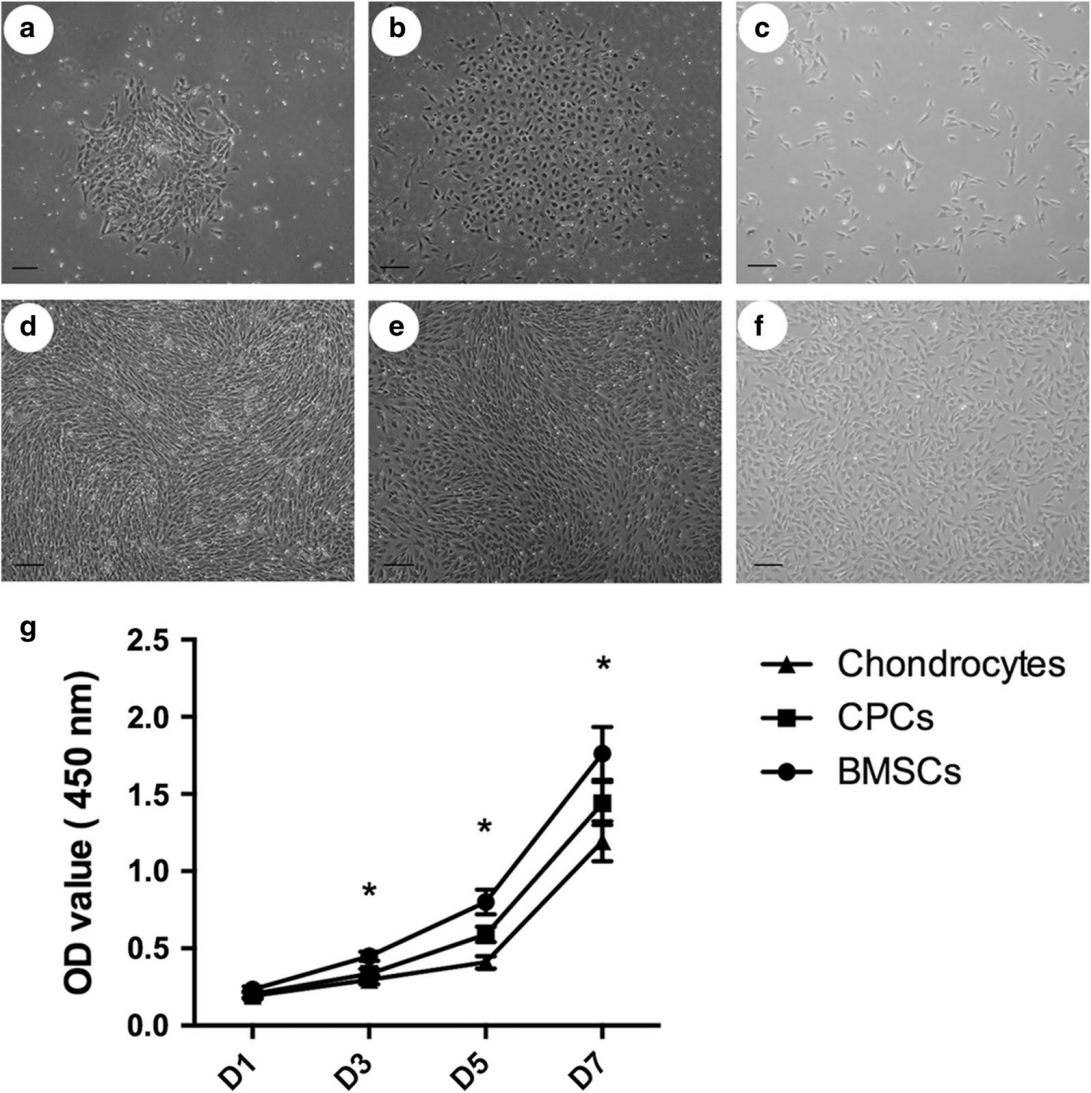
Cell culture in vitro. BMSCs (**a**) and CPCs (**b**) were found to form colonies after 2 weeks of primary culture, while chondrocytes were uniformly distributed (**c**). BMSCs (**d**) and CPCs (**e**) at passage 1 were arranged in whorls or bundles, while chondrocytes (**f**) were polygonal. (**g**) There was a significant difference in proliferation among these three cell populations (*p < 0.05)

Colony formation was observed after 2 weeks of primary cultures of CPCs and BMSCs. The articular cartilage-derived stem/progenitor cells were small, rounded and polygonal in primary culture; the BMSCs were spindle shaped in primary culture; while chondrocytes were polygonal. CPCs and BMSCs at passage 1 had a fibroblast-like fusiform shape and were arranged in whorls or bundles upon reaching 80–90% confluence, while the chondrocytes remained polygonal (Fig. 1).

### Cell proliferation

To determine the cell proliferation capability, the proliferative rates were analysed using a CCK-8 assay. There was a significant difference in proliferation between CPCs and BMSCs, as well as between chondrocytes and CPCs (p < 0.05); specifically, BMSCs showed a higher proliferative ability than CPCs, and CPCs had a higher proliferative potential than chondrocytes (p < 0.05) (Fig. 1).

### Characteristics of scaffold and cell-scaffold constructs

**Fig. 2:**
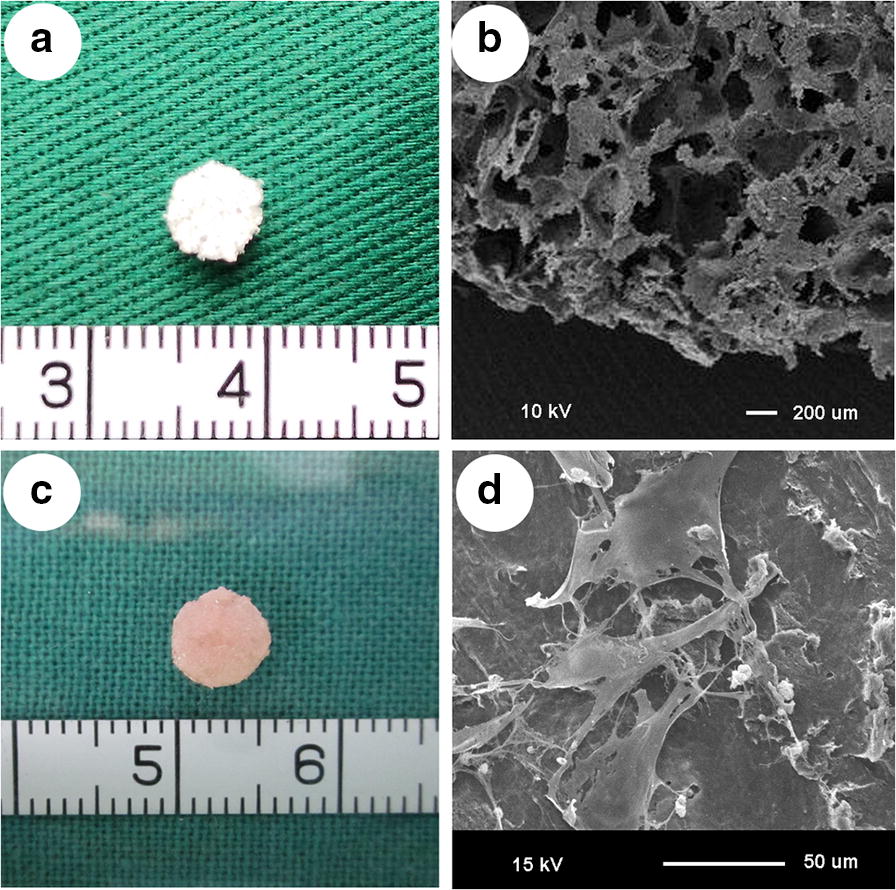
Preparation of scaffold and cell-scaffold constructs. **a** PHBV scaffolds were cut into cylinder shapes (5 mm diameter, 2 mm thickness). **b** SEM revealed the macro-porous structures of the PHBV scaffolds. **c** Good compatibility between the cells and the scaffold was observed on day 7. **d** SEM data demonstrated that CPCs adhered to the surface of the scaffold after 1 week of in vitro culture

**Fig. 3:**
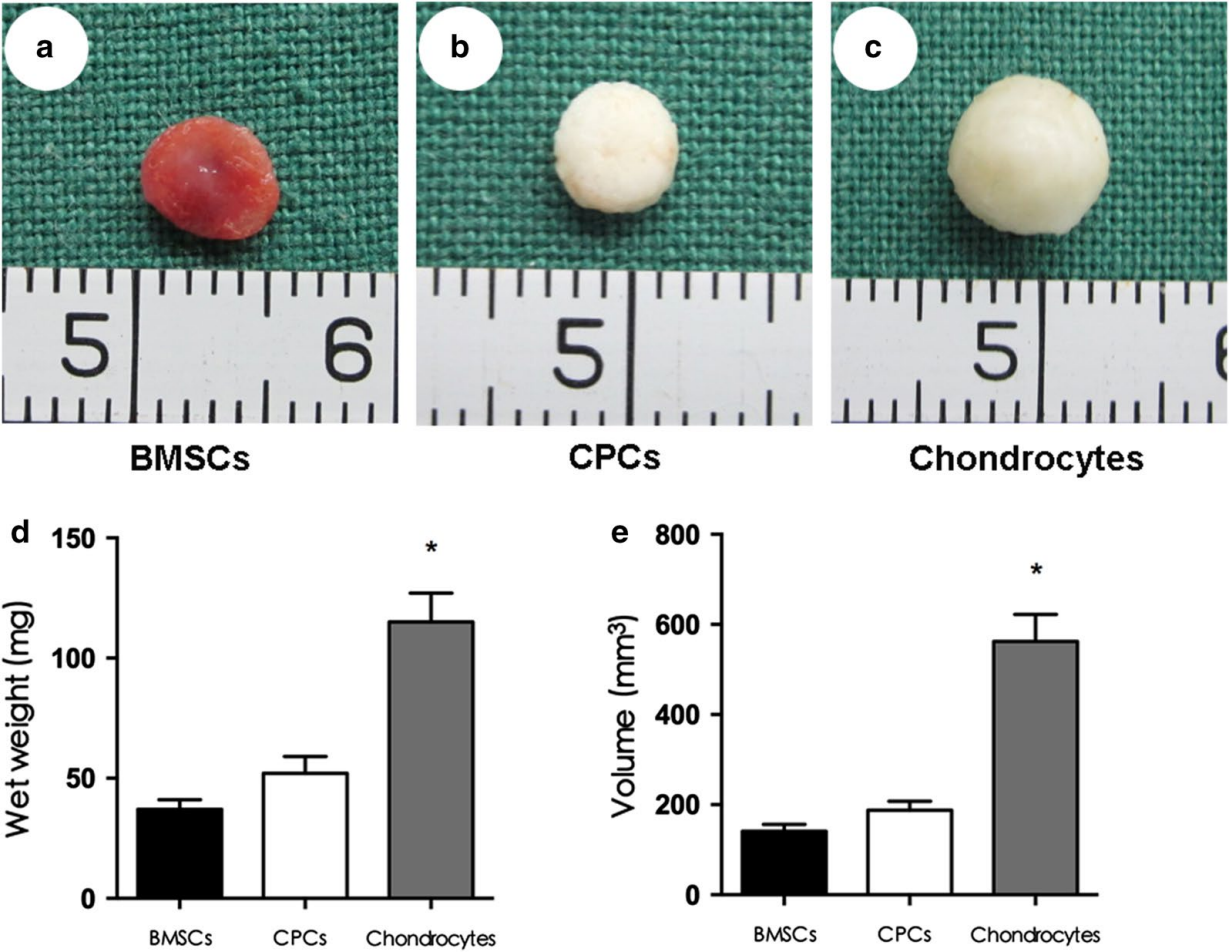
Properties of the cell-scaffold constructs 6 weeks after implantation. **a** The BMSCs-scaffold constructs became obviously vascularized tissue 6 weeks after subcutaneous transplantation. **b** The CPCs-scaffold constructs became an ivory-whitish tissue, and **c** the chondrocyte-PHBV constructs became a cartilage-like tissue. There was a significant difference among these three groups in the measured wet weight (**d**) and volume (**e**) (*p < 0.05)

As shown in Fig. 3, the PHBV scaffolds were cut into cylinders of the same size (5 mm side diameter, 2 mm thickness). SEM data demonstrated that the PHBV scaffolds had macro-porous structures with interconnected open pores, and the pore size varied from 30 mm to 300 mm (Fig. 2).

Gross observation and SEM analysis show the good compatibility between the cells and the scaffold and the production of considerable amounts of extracellular matrix (ECM) after 1 week of in vitro culture (scale bar = 100 mm).

### Gross view, wet weight, and volume of in vivo cell-scaffold constructs

After 6 weeks of culture in vivo, the BMSC-scaffold constructs became obviously vascularized, and the CPC-scaffold constructs roughly maintained their original size and shape and became an ivory-whitish tissue. The constructs in the chondrocyte group became cartilage-like tissue (Fig. 3).

The wet weight and volume measurement showed a significant difference among these three groups (p < 0.05), and the measured wet weight and volume in the chondrocyte group were much higher than those in the BMSC and CPC groups, indicating that chondrocytes produce a large amount of ECM (p < 0.05).

### Histological and immunohistochemical staining

**Fig. 4:**
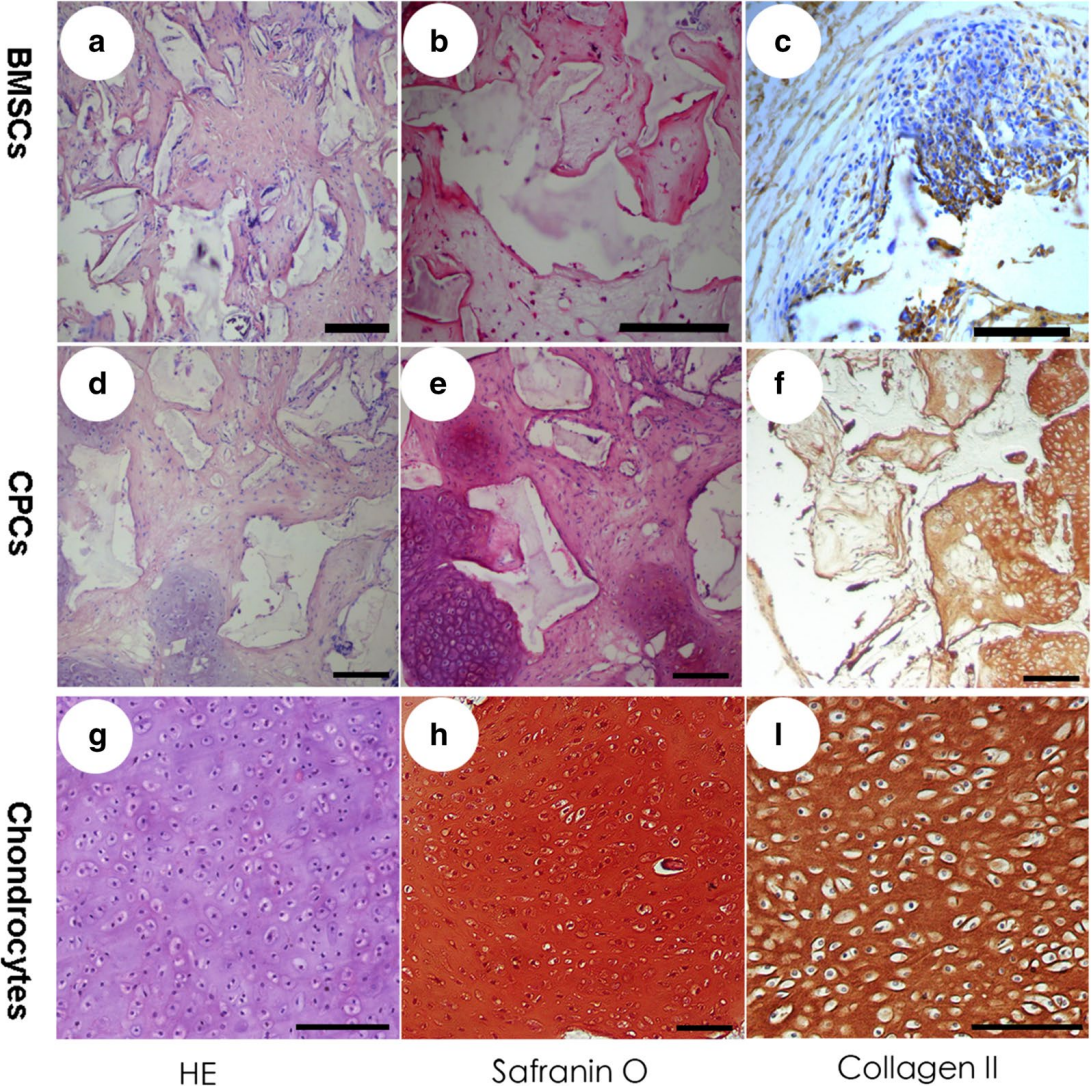
Histological and immunohistochemical staining. Haematoxylin and eosin (HE) staining showed that no cartilage structure in the BMSCs group was observed after 6 weeks of in vivo culture (**a**), while the formation of a typical cartilage structure was observed in the CPCs group (**d**) and the chondrocyte group (**g**). No expression of safranin-*O* (**b**) and COL II (**c**) was observed in the BMSC group, some cells were positive for safranin-*O* (**e**) and COL II (**f**) in the CPCs group, and strong positive expression of safranin-O (**h**) and COL II (**i**) was observed in the chondrocyte groups

In the current study, histological examination showed the formation of a typical cartilage structure after 6 weeks of in vivo culture in the chondrocyte and CPC groups, while no cartilage structure was observed in the BMSC group. These findings were further supported by immunohistochemical staining. Strong positive expression of COL II was observed in the chondrocyte groups, some positive expression of COL II was observed in the CPC group, while no expression of COL II was observed in the BMSC group. These results indicate that CPCs underwent spontaneous chondrogenic differentiation without chondrogenic induction, while BMSCs could not achieve a chondrogenic differentiated stage without chondrogenic induction (Fig. 4).

### Biomechanical and biochemical properties of in vivo cell-scaffold constructs

Six weeks after in vivo transplantation, the total collagen content and the GAG content in the chondrocyte group were higher than those in the BMSC and CPC groups, and the total collagen content and GAG content in the CPC group were higher than those in the BMSC group (p < 0.05).

**Fig. 5:**
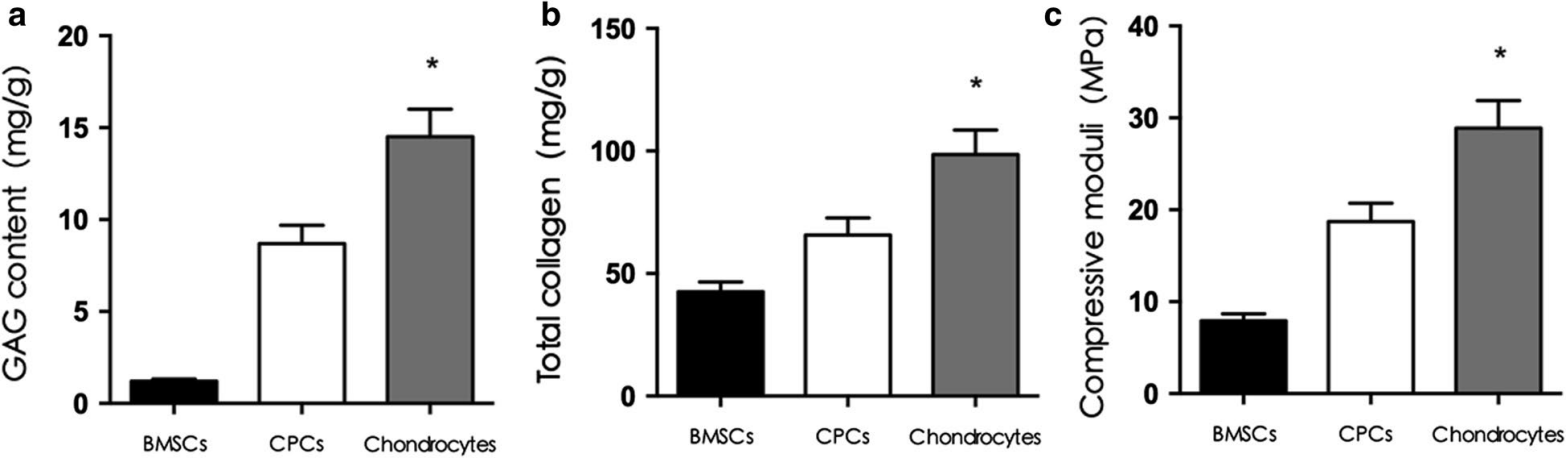
Biomechanical and biochemical properties of the cell-scaffold constructs in vivo. There was a significant difference in total collagen content and GAG content, as well as compressive moduli among the three groups 6 weeks after in vivo transplantation (*p < 0.05)

These findings were further supported by mechanical strength measurements. Six weeks after in vivo transplantation, the compressive modulus of the BMSC-PHBV scaffold was 7.9 ± 1.1 MPa, the compressive modulus of the CPCs-PHBV scaffold was 18.7 ± 2.3 MPa, the compressive modulus of the chondrocyte-PHBV scaffold was 28.9 ± 4.2 MPa, and there was a significant difference among the three groups (p < 0.05) (Fig. 5).

### Gene expression

**Fig. 6:**
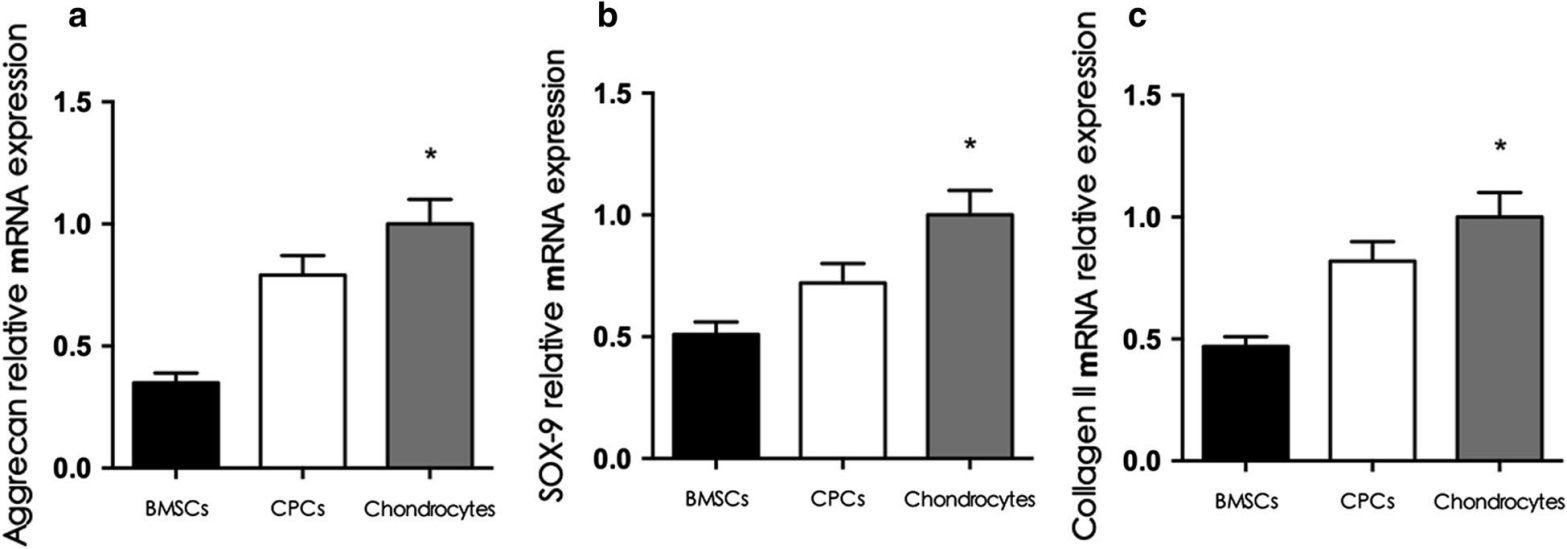
Chondrogenic gene expression. RT-PCR data showed that the relative expression of sox-9, collagen II, and aggrecan in the chondrocyte group was higher than that in the CPCs group (p < 0.05), and the relative expression of these genes in the CPCs group was higher than that in the BMSCs group (p < 0.05)

The relative expression of chondrogenic genes (sox-9, collagen II, and aggrecan) in the chondrocyte group was higher than that in the BMSC and CPC groups, and the relative expression of sox-9, collagen II, and aggrecan in the CPC group was higher than that in the BMSC group (p < 0.05) (Fig. 6).

### VEGF expression of cell-PHBV constructs

**Fig. 7:**
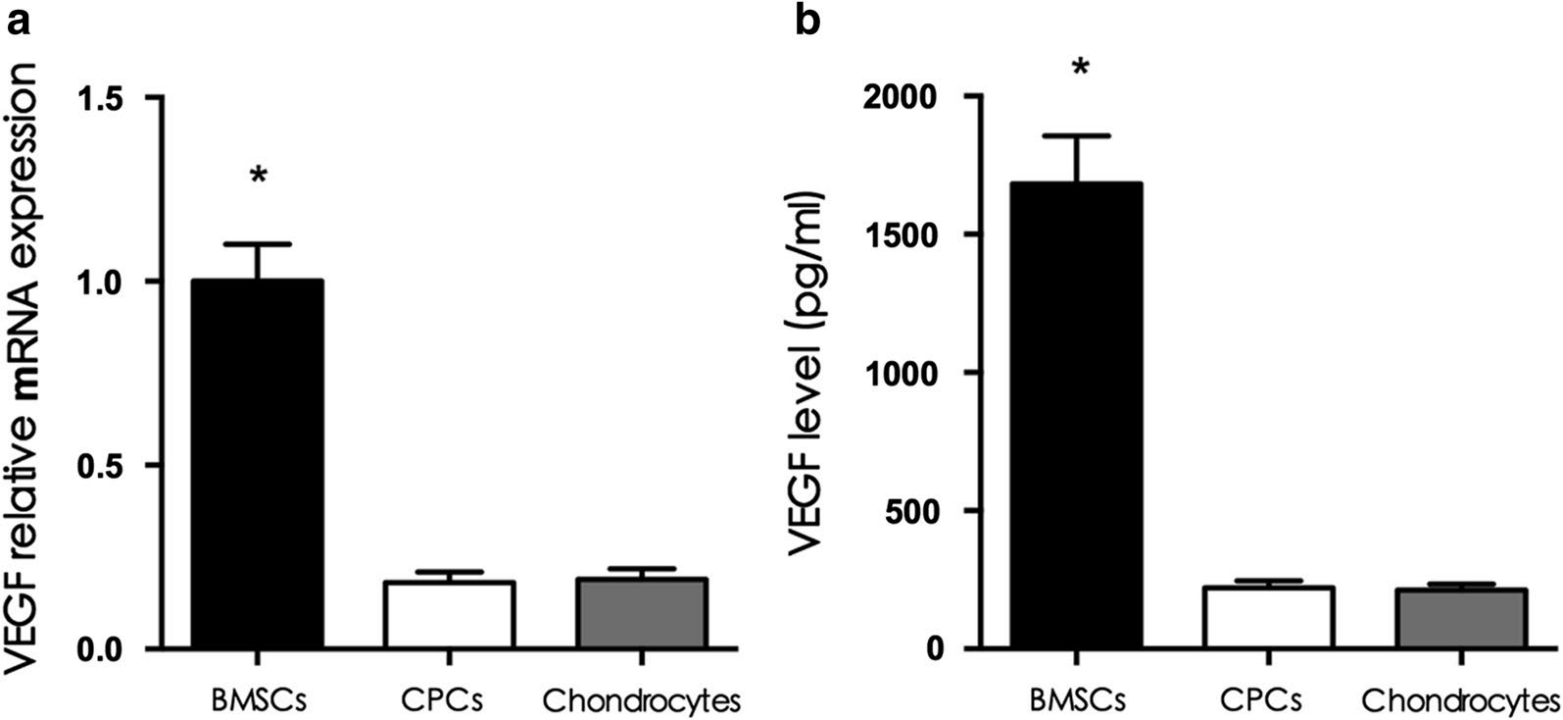
VEGF expression of the cell-PHBV constructs. The VEGF mRNA expression and protein content were higher in the BMSCs-PHBV construct group than in the CPCs-PHBV construct group and the chondrocyte-PHBV construct group (p < 0.05), while there was no significant difference between the CPCs-PHBV construct group and the chondrocyte-PHBV construct group (p > 0.05)

To evaluate vascularization after transplantation, the VEGF mRNA expression and protein content were determined. A significant increase in VEGF mRNA expression and protein content was observed for the BMSC-PHBV constructs compared with the CPC-PHBV constructs and the chondrocyte-PHBV constructs, demonstrating that vascular invasion occurred in the BMSC-PHBV constructs (p < 0.05; Fig. 7).

## Discussion

Due to the poor self-repair and regeneration capacity of cartilage, the treatment of cartilage defects is a knotty problem, and satisfactory therapeutic options are very scarce [26]. Advances in biomedicine and biomaterials have promoted the development of new cartilage repair techniques, and cartilage tissue engineering provides a novel alternative therapeutic option for the regeneration of cartilage tissue that is damaged due to trauma or disease [27].

The basic approach of cartilage tissue engineering involves the application of cells, scaffolds, and a specific microenvironment alone or in combination [28]. As a key element in cartilage tissue engineering, seeding cells play a vital role [29]. The major challenges in cartilage engineering include selection of the seeding cell source, in vitro expansion, and differentiation [30, 31]. Chondrocytes, BMSCs, and adipose-derived stem cells (ADSCs), as well as other cells have all been explored for their potential as an ideal cell source for cartilage regeneration [27, 28]. Chondrocytes, the predominant cell type in cartilage, synthesize matrix components and were the first seeding cells used in cartilage tissue engineering because chondrocytes are the only cell found in native cartilage, while the poor proliferation ability and the dedifferentiation of chondrocytes are bottlenecks in the clinical application of chondrocytes [32]. Recently, there has been increasing interest in stem cell-based cartilage tissue engineering options in surgical practice to deal with lost or damaged cartilage tissue, and BMSCs could be promising cell sources for use in cartilage regeneration [21]. BMSCs have multipotent differentiation potential and high proliferation potential, and an increasing number of studies have demonstrated that chondrogenically differentiated BMSCs underwent vascularization or endochondral ossification after in vivo transplantation [10, 20].

Cartilage progenitor cells, which are characterized by stem cell markers, multi-lineage ability, and their self-renewal potential, have recently been found in different cartilage tissues, such as auricular cartilage, articular cartilage, and nasal cartilage, and these stem/progenitor cells are thought to respond to injury and migrate into cartilage defect zones [33, 34]. Therefore, in the current study, we explored the feasibility of combining CPCs with PHBV to produce tissue-engineered cartilage and compared the proliferation ability and chondrogenic characteristics CPCs with those of BMSCs and chondrocytes.

Cartilage tissue engineering requires a considerable number of seeding cells, and the proliferative ability of seeding cells plays a vital role in cartilage tissue engineering [35]. It has been reported that mature chondrocytes that have reached the end of the differentiation process do not have the capacity to proliferate or differentiate [36]. Chondrocytes are currently considered terminally differentiated cells and thus represent the last stage of differentiation in the chondrogenic cell lineage, and terminally differentiated chondrocytes have a limited proliferative capacity [37]. In addition, in vitro expansion of chondrocytes leads to dedifferentiation of mature chondrocytes, which is characterized by increased expression of type I collagen, decreased expression of COL II and decreased proteoglycan content. In contrast, BMSCs have been reported to be an undifferentiated population capable of endless self-renewal and have high proliferative potential [38]. In the current study, we first compared the proliferation characteristics of chondrocytes, BMSCs, and CPCs. The current results indicated that CPCs have a higher proliferation rate than chondrocytes and a lower proliferation rate than BMSCs.

Chondrogenic differentiation potential is an important index of stem cells [39]. Many studies have presented the advantages and feasibility of using BMSCs to treat cartilage defects [40]. For chondrogenic differentiation of BMSCs, the incomplete chondrogenesis and the formation of fibrocartilage remain difficult problems [41]. During the process of chondrogenesis, BMSCs adopt a transient chondrocyte phenotype rather than a permanent state and tend to undergo terminal differentiation, which is followed by endochondral ossification [10]. In our previous study, we also found that some chondrogenic-induced human BMSCs in vitro became ossified after implantation in vitro [41].

The key problem of neo-cartilage tissue regenerated by mesenchymal stem cells is the failure to maintain the chondrocyte phenotype: on the one hand, the cell origin may determine the ultimate fate of the mesenchymal stem cell (MSC) regenerated cartilage tissue [10]; on the other hand, MSCs tend to lose their chondrogenic properties simultaneously upon persistent exposure to the chondrogenic stimuli, such as TGF- β and dexamethasone, prevalent in current culture methods, indicating that exogenous factors may lead to loss of the chondrogenic phenotype and may promote chondrocyte hypertrophy and endochondral ossification [42]. To prevent exogenous chondrogenic stimuli from affecting the chondrogenic differentiation potential, we combined the chondrocytes, BMSCs, and CPCs with the biomaterial-PHBV separately without chondrogenic induction and implanted the combination subcutaneously, and we found that the tissue formed by BMSCs without induction became obviously vascularized 6 weeks after implantation, while the cartilage engineered by chondrocytes without induction exhibited a mature cartilage appearance 6 weeks after implantation. These findings were further supported by the histology and immunohistochemistry results. More interestingly, we found that the tissue formed by CPCs without induction formed cartilage-like tissues with an ivory-whitish appearance, indicating that CPCs could differentiate into chondrocytes spontaneously without chondrogenic induction. Normal chondrocytes express high levels of COL II and aggrecan, and stem or progenitor cells express chondrocyte-specific genes only in the presence of specific induction conditions (TGF-β1 (10 ng/ml), FGF (25 ng/ml), ITS (1 : 100), and dexamethasone (10–7 M), without FBS) [43, 44]. We also found that cartilage-derived stem cells express chondrocyte-specific genes without specific induction medium containing TGF-β1 and dexamethasone. There are two possible explanations for the spontaneous chondrogenesis of CPCs: on the one hand, BMSCs have been reported to be multipotent progenitor cells because of their capability to differentiate into several mesenchymal cells, including osteoblasts, adipocytes, tenocytes, fibroblasts, and myoblasts, rather than just chondrocytes. On the one hand, the differentiation potential of CPCs, may be mainly confined to chondrogenesis. On the other hand, CPCs are isolated from chondrocytes by a differential adhesion assay to fibronectin, and the so-called “CPCs” may be a mixture of progenitor cells and chondrocytes, not pure CPCs. CPCs may undergo spontaneous chondrogenic differentiation without chondrogenic induction, while BMSCs cannot reach a chondrogenic differentiation stage without chondrogenic induction.

## Conclusion

The current study indiactes that CPCs can be easily isolated, are capable of expansion, and can be cultured to express and synthesize cartilage-specific molecules. In addition, CPCs may overcome the “dedifferentiation” of chondrocytes, and “vascularization or ossification” of BMSCs, and then become the ideal seeding cells for cartilage tissue engineering.

## Abbreviations

ACI: autologous chondrocyte implantation
OA: osteoarthritis
BMSC: bone mesenchymal stem cell
CPCs: cartilage progenitor cells
PHBV: poly(3-hydroxybutyrate-co-3-hydroxyvalerate)
CCK: cell counting kit
GAG: glycosaminoglycan
HE: haematoxylin and eosin
COL II: type II collagen
RT: reverse transcription
ECM: extracellular matrix
ADSCs: adipose-derived stem cells
MSC: mesenchymal stem cell
